# The Rate of Decline of Sugarbeet Cyst Nematode in Central California Under Nonhost Crops May Impact Biofuel Production

**DOI:** 10.2478/jofnem-2024-0045

**Published:** 2024-11-10

**Authors:** B. B. Westerdahl, E. P. Caswell-Chen, F. R. Kegel

**Affiliations:** University of California, Department of Entomology and Nematology, Davis, CA 95616; University of California, Cooperative Extension, Stockton, CA 95206

**Keywords:** *Beta vulgaris*, bioethanol, biofuel, crop rotation, *Heterodera schachtii*, pest management, population decline, sugarbeet

## Abstract

Crop rotation has been a commercial practice for managing the sugarbeet cyst nematode (*Heterodera schachtii*, SBCN) since the 1950s. Research conducted in southern California established that SBCN populations decline at the rate of 49% to 80% per year, leading to estimates that three- to four-year rotations to nonhost crops would be sufficient to reduce SBCN densities to nondamaging levels. Following grower reports that much longer rotations were needed in central California, trials were conducted to establish the rate of decline of SBCN in the San Joaquin Valley. Ten commercial fields with a history of SBCN infestation were sampled periodically for up to 6.3 years. In each field, 10 circular subplots located 30.5 meters apart (each with a 6-m radius) were established with reference to a permanent landmark. On each sampling date, 12 subsamples from each subplot were taken randomly from the top 0 cm to 30 cm of soil and composited into a single sample. Standard techniques were utilized to extract and count cysts and eggs from soil samples. Average yearly rates of population decline in the sampled fields ranged from 11.4% to 25.8%. This finding has implications for SBCN management in California sugarbeets grown for biofuel, as the lower decline rates indicate that longer nonhost rotation periods than previously anticipated may be necessary.

In California, sugarbeets (*Beta vulgaris*) have been grown across the state, from the northern Klamath Basin to the southern Imperial Valley (Goodwin et al., 1998). In recent years, the industry has seen a dramatic decline in hectares and processing facilities. Causes of this decline include pests and diseases, urbanization, conversion of row crop land to trees and vines, and low sugar prices. The primary pests are the sugarbeet cyst nematode (*Heterodera schachtii*, SBCN), *Rhizomania* (caused by Beet necrotic yellow vein virus, which is transmitted by the root-infecting parasite *Polymyxa betae*), and the aphid-transmitted virus yellows (Goodwin et al., 1998).

SBCN is widespread in the Sacramento, San Joaquin, and Imperial Valleys. In 1978, more than 80,940 ha in California were infested with SBCN (Roberts and Thomason, 1981). This nematode is thought to have been brought into California many years ago and to have been accidentally distributed throughout much of the sugarbeet growing area. Because of its importance on sugarbeet and cole crops, considerable research on SBCN has been conducted in California (Baldwin and Mundo-Ocampo, 1991; Burt and Ferris, 1996; Caswell and Thomason, 1991; Gardner and Caswell-Chen, 1993, 1997; Lear et al., 1966; Raski and Johnson, 1959; Roberts, 1985; Roberts and Thomason, 1981; Roberts et al., 1981; Steele, 1984).

In SBCN, the second-stage infective juvenile hatches from the egg, is attracted to host roots by exudates, penetrates a host root, and establishes a permanent feeding site. The nematode feeds and grows to the adult stage, with the adult female retaining most of the eggs (up to 600) internally. The female body hardens after death, protecting the eggs from adverse environmental conditions (Roberts and Thomason, 1981). Activity, reproduction, and development occur between 8°C and 35°C, and reproduction is most rapid between 21°C and 27°C (Thomason and Fife, 1962; Caswell and Thomason, 1991). The developmental periods from J2 to J3, J4, adult, and the next generation (J2) are 100, 140, 225, and 399 degree-days (base 8°C), respectively (Griffin, 1988; Caswell and Thomason, 1991). In the absence of a host, cysts containing eggs persist in the soil for many years. Although the presence of host roots stimulates an egg hatch, a certain number of eggs hatch each year — even in the absence of a host — resulting in a slow decrease in viable eggs.

Historically, both crop rotation and nematicides have been used to control SBCN (Altman and Thomason, 1971; Cooke, 1993). Roberts et al. (1981) found that annual decline rates of SBCN in the Imperial Valley ranged from 49% to 56% and 63% to 80% at 0 to 30- and 30 to 60-cm-depths, respectively. They developed a model to predict lengths of rotation needed for nonhost crops. Burt and Ferris (1996) examined economic consequences of various rotation crops, with decline rates ranging from 50% to 75% a year. Our study was initiated because growers and University of California farm advisors in the San Joaquin Valley reported needing longer lengths of rotation (F. Kegel, personal communication) than predicted by previous research conducted by Roberts et al. (1981) in southern California; decline rates of 49% to 56% per year corresponded to a need for three to four years of rotation.

Throughout California, diversity in planting and harvest dates directly impacts the population dynamics of SBCN. In southern California, planting dates in the Imperial Valley are September through October, with harvest in April through July. In central California, the Sacramento and San Joaquin Valleys established two planting cycles referred to as “spring harvest” and “fall harvest.” “Spring harvest” sugarbeets would be planted in May or June and harvested April through June the following year. “Fall harvest” sugarbeets would be planted January through April and harvested September through October of the same year. The San Joaquin Valley established an additional “summer harvest,” with planting in October through January and harvest in July or August. Geographical boundaries for these planting cycles were developed by joint agreement between the sugarbeet processors and the California Beet Growers Association. The desire to maximize the length of time processing plants operate each year was the primary reason for the multiple planting and harvest dates. In the Sacramento and San Joaquin Valley areas, the rainy season typically extends from October to April or later, which prevents the harvesting of sugarbeets. The fine-textured soils in which sugarbeets grow remain wet until late spring, preventing harvesting. The desire to minimize transmission of aphid-transmitted virus yellows from older to younger plantings was another reason to establish geographical boundaries for the different planting dates (Goodwin et al., 1998).

Because the life history of SBCN is highly temperature dependent, the variability in planting and harvesting dates could result in variable reproduction during a growing season, with subsequent variability in the lengths of rotation needed between nonhost crops. This research was conducted to establish the decline rate of SBCN in spring- and fall-harvested fields in the San Joaquin Valley to help predict the length of rotation needed for sugarbeets.

In recent years, there has been increasing interest in using sugarbeets for production of ethanol for use as a biofuel. European countries produce ethanol from sugarbeets (Flach et al., 2020; Marzo et al., 2019; Voegele, 2019, 2020), and production economics have been evaluated in the United States (Haankuku et al., 2015). Trial runs producing ethanol from sugarbeets have been made in California (Schill, 2015) and North Dakota (Thompson, 2019). The economic potential for bioethanol production from sugarbeets grown in California has been assessed (Alexiades et al., 2017; Panella, 2010; Panella and Kaffka, 2010). Transportation fuels sold in California are regulated by the state’s Low Carbon Fuel Standard (LCFS) and the federal Renewable Fuels Standard. Factors determined to be favorable include financial rewards from the state’s LCFS, yearly increases in yields, potential for year-round harvesting, and new technologies to convert sugarbeets to ethanol. Most sugarbeet production areas in the United States have fields infested with SBCN, which causes significant yield reductions. Rotation to nonhost crops for multiple years is a recommended management option. The length of rotation is determined by the expected rate of SBCN egg decline following the harvest of sugarbeets. The potential impact of the length of rotation to nonhost crops could be a significant factor in the economics of using sugarbeets for the production of biofuel. A slower rate of decline would indicate less frequent cropping to sugarbeets to be used as a biofuel.

## Materials and Methods

Ten commercial fields in the San Joaquin Valley with a history of SBCN infestation were selected. Eight were in a “spring harvest” area (Fields A to H) known as Collegeville (N37°54.285′, W121°08.830″), and two were in a “fall harvest” area (Fields I to J) known as Eight Mile Road (N38°03.775″, W121°23.514′). In each field, 10 circular subplots located 30.5 meters apart (each with a 6-m radius) were established with reference to a permanent landmark. On each sampling date, 12 subsamples from each subplot were taken randomly with a shovel from the top 0 to 30 cm of soil and composited into a single sample of approximately 1 kg. Standard techniques were utilized to extract and count cysts from soil and eggs (Caswell et al., 1985; McKenry and Roberts, 1985). The extraction method is described in detail by Caswell et al. (1985). Briefly, 350 g of soil is air dried in paper bags. The soil is then thoroughly mixed, and cysts are separated from the soil using a modified Fenwick flotation can and caught on a 150-μm sieve. Cysts are washed onto tissue paper and air dried. Dried cysts are floated off the tissue onto filter paper in an ethanol-glycerine flotation apparatus and counted. Eggs are released from cysts using a tissue homogenizer (CEKA, Type UM, E. Bùhler, Tübingen, Germany) and then counted.

The standard extraction procedure for SBCN requires soil to be thoroughly dried prior to extraction. Because drying is not conducive to extraction of other nematode species, on the first sampling date for each field, nematodes were also extracted via elutriation followed by centrifugation (Byrd et al., 1976) to determine other nematodes which might be present. Fields were sampled approximately yearly for up to 6.3 years. Growers followed their normal crop rotation sequences which included corn (*Zea mays*), tomato (*Lycopersicon esculentum*), beans (*Phaseolus vulgaris*), wheat (*Triticum vulgare*), cabbage (*Brassica oleracea*), or Bok choy (*Brassica rapa*). A composite soil sample was analyzed by the University of California DANR Analytical Laboratory for physical and chemical properties. Data were evaluated by analysis of variance followed by Duncan’s New Multiple Range Test and regression (JMP, SAS Institute Inc.).

## Results

Field A was in cabbage when first sampled. Rotation crops included tomatoes (three times), cabbage, and wheat (two times) (Table 1). During the 6.3 years that populations were followed in this field, there was an overall decline from 4.35 eggs and 0.267 cysts to 0.41 eggs and 0.067 cysts per gram of soil, in spite of having cabbage in the rotation (Table 2). Eggs per cyst declined from 10.9 to 2.1. Between 2.8 and 3.8 years after first sampling, a slight but significant increase in cysts was noted when the field was cropped to tomatoes (*P* ≤ 0.05). The number of eggs and cysts more than doubled during this time. The yearly rate of egg decline in this field was 12.2% (*y* = 1.11005e – 0.29642*x*, *r*^2^ = 0.702291, *P* ≤ 0. 0.0372, where *x* = years and *y* = eggs/gram of soil).

**Table 1:** Cropping history, field size, and nematodes present other than sugarbeet cyst nematode for the 10 San Joaquin Valley fields (A to J) sampled in the study.

**Years**	**Field Location**									
	**A**	**B**	**C**	**D**	**E**	**F**	**G**	**H**	**I**	**J**
−1^a^	Cabbage	Bok choy	Wheat	Sugarbeet	Sugarbeet	Sugarbeet	Sugarbeet	Sugarbeet		Sugarbeet
0	Cabbage	Bok choy	Sugarbeet	Bean	Bean	Tomato	Tomato	Bean	Sugarbeet	Tomato
1	Tomato	Beans	Beans	Wheat	Wheat	Wheat	Wheat	Wheat	Corn	Corn
2	Cabbage	Cabbage	Tomato	Fallow	Tomato	Tomato	Tomato	Tomato	Fallow	Corn
3	Wheat	Wheat	Wheat	Bean	Tomato	Tomato	Tomato	Tomato	Sugarbeet	Corn
4	Tomato	Tomato	Tomato	Wheat	Wheat	Wheat	Wheat	Wheat	Fallow	
5	Wheat	Tomato	Tomato	Corn	Tomato	Tomato	Tomato	Tomato	Fallow	
6	Tomato	Beans	Wheat	Bean	Wheat	Wheat	Wheat	Wheat		

Size of field (ha)	32	3	32	32	8	8	8	8	32	32

Years out of sugarbeet prior to −1	Unknown	8	7	Unknown	4	4	8	8	4	20

Other plant-parasitic nematodes	none detected	none detected	*Pratylenchus* sp.	*Tylenchorhynchus* sp.	*Meloidogyne* sp., *Pratylenchus* sp., *Xiphinema* sp.	*Meloidogyne* sp., *Pratylenchus* sp., *Xiphinema* sp.	none detected	none detected	none detected	*Helicotylenchus* sp.

a Crop in field year prior to first sampling date.

Field B had not been planted to sugarbeets for approximately eight years prior to first sampling but had been planted to Bok choy — a host of SBCN — for two years prior to first sampling. Bok choy is a 60- to 90-day crop and was the only one planted each year. Rotation crops included beans (two times), cabbage, wheat, and tomatoes (two times) (Table 1). Cysts, eggs, and the number of eggs per cyst were relatively low at the time of first sampling and remained at low levels until 3.8 years when the field was planted to tomatoes (Table 2). At this time, a significant increase in cysts, eggs, and eggs per cyst occurred (*P* ≤ 0.05). A regression equation fit on the sampling points prior to planting tomatoes indicates a yearly population decline of 34.6% for eggs (*y* = 0.10491 − 0.03632*x*, *r*^2^ = 0.390959, *P* ≤ 0.1049054, where *x* = years and *y* = eggs/gram of soil).

At the time of first sampling, field C was just about to be planted to sugarbeets for the first time in eight years. Populations increased significantly under beets (*P* ≤ 0.05) and then declined during subsequent years (Table 2). Rotation crops included beans, tomatoes (three times), and wheat (two times) (Table 1). During the second planting of tomatoes at 3.8 years, a significant increase (*P* ≤ 0.05) in cysts but not eggs occurred. A regression line fit through all points except the first, which demonstrated a yearly decline rate for eggs of 12.4% (*y* = 0.76579e − 0.24778*x*, *r*^2^ = 0.580384, *P* ≤ 0.1344, where *x* = years and *y* = eggs/gram of soil).

**Table 2:** Recovery of *Heterodera schachtii* eggs and cysts from 10 San Joaquin Valley fields for multiple years after initiation of sampling.

Field Location		Number/gram					

	Years	Eggs		Cysts		Eggs/cyst	
A	0.0	4.35	b^a^	0.267	c	10.9	c
	0.3	1.88	a	0.114	abc	7.6	bc
	1.8	2.07	a	0.134	abc	8.7	bc
	2.8	0.74	a	0.034	a	5.3	ab
	3.8	1.80	a	0.223	bc	3.5	ab
	6.3	0.41	a	0.067	ab	2.1	a
B	0.0	0.17	a	0.011	a	1.2	a
	0.5	0.02	a	0.001	a	0.2	a
	1.8	0.01	a	0.001	a	0.1	a
	2.8	0.03	a	0.002	a	0.3	a
	3.8	2.94	b	0.187	b	5.4	b
	5.1	0.21	a	0.038	a	1.4	a
C	0.0	0.21	a	0.029	a	1.4	a
	1.8	1.78	b	0.150	bc	7.0	b
	2.8	0.65	a	0.052	a	4.2	a
	3.8	1.27	ab	0.168	c	3.4	a
	5.1	0.47	a	0.072	ab	2.0	a
	6.3	0.51	a	0.097	abc	2.4	a
D	0.0	1.28	ab	0.036	a	6.3	bc
	0.3	0.59	ab	0.066	a	2.2	ab
	1.8	1.44	b	0.072	a	7.0	bc
	2.8	1.03	ab	0.038	a	7.9	c
	4.4	0.31	ab	0.019	a	2.5	ab
	6.3	0.13	a	0.026	a	0.9	a
E	0.0	1.08	c	0.066	c	6.1	d
	0.3	0.84	bc	0.044	bc	5.2	bcd
	1.8	0.72	abc	0.025	ab	5.7	cd
	2.8	0.23	ab	0.019	ab	2.0	abc
	4.4	0.12	a	0.003	a	1.1	a
	5.1	0.31	ab	0.013	ab	2.7	abcd
	6.3	0.21	ab	0.032	ab	1.3	ab
F	0.0	1.60	b	0.089	ab	6.1	b
	0.3	0.90	ab	0.127	b	3.9	ab
	1.8	1.14	ab	0.059	ab	5.6	ab
	2.8	0.32	ab	0.049	ab	1.9	ab
	4.4	0.10	a	0.018	a	0.7	a
	5.1	0.35	ab	0.061	ab	2.0	ab
	6.3	0.06	a	0.019	a	0.4	a
G	0.0	0.04	a	0.002	ab	0.3	a
	0.3	0.15	a	0.009	bc	1.2	a
	1.8	0.00	a	0.001	a	0.0	a
	2.8	0.13	a	0.005	abc	1.0	a
	4.4	0.03	a	0.001	ab	0.3	a
	5.1	0.09	a	0.013	c	0.8	a
	6.3	0.00	a	0.000	a	0.0	a
H	0.0	0.00	a	0.006	bc	0.0	a
	0.3	0.67	b	0.006	bc	6.4	b
	2.0	0.18	a	0.001	a	1.8	a
	2.8	0.01	a	0.010	c	0.1	a
	4.4	0.00	a	0.001	ab	0.0	a
	5.1	0.12	a	0.003	ab	1.2	a
	6.3	0.00	a	0.001	ab	0.0	a
I	0.0	9.59	c	0.204	a	25.0	b
	1.8	7.00	bc	0.230	a	20.8	b
	2.3	3.99	ab	0.121	a	16.5	b
	3.3	1.72	a	0.155	a	4.0	a
	4.6	1.17	a	0.151	a	4.7	a
J	0.0	1.51	b	0.188	b	5.1	a
	0.3	1.38	b	0.172	b	5.2	a
	2.8	0.40	a	0.042	a	3.0	a
	3.8	0.32	a	0.074	a	1.9	a

Data are means of 10 replicates.

a = significantly different from first sample in that field at *P* ≤ 0.05, according to Duncan’s New Multiple Range Test.

Field D was in sugarbeets at the time of first sampling. Rotation included fallow, beans (three times), corn, and wheat (two times) (Table 1). Populations of eggs and cysts did not change significantly during the course of the study (*P* ≤ 0.05) (Table 2). An increase in the number of eggs per cyst (*P* ≤ 0.05) occurred during a year when the field was fallow (1.8 to 2.8 years). A regression line fitted through all sampling points indicated a yearly decline in eggs of 20.7% (*y* = 0.30158e − 0.3138x, *r*^2^ = 0.682169, *P* ≤ 0.0428, where *x* = years and *y* = eggs/gram of soil).

Fields E, F, G, and H were planted to sugarbeets the year prior to the first sampling and were farmed as a single unit during that time. One half of the field (G and H) had not previously been in sugarbeets for eight years. The other half of the field (E and F) had been planted to sugarbeets four years earlier. Following harvest of the sugarbeets, the grower elected to divide the field perpendicular to the original division and followed a different cropping pattern in fields E and H than in fields F and G (Table 1). Rotation crops in E and H were beans, tomatoes (three times), and wheat (three times). Rotation crops in F and G were tomatoes (four times) and wheat (three times).

During the 6.3 years that populations were followed in E, there was an overall decline from 1.08 eggs and 0.066 cysts to 0.21 eggs and 0.032 cysts per gram of soil (*P* ≤ 0.05) (Table 2). The number of eggs per cyst declined from 6.1 to 1.3 (*P* ≤ 0.05) (Table 1). A regression line fitted through all sampling points indicated a yearly decline in eggs of 15% (*y* = −0.135*x* + 0.8993, *r*^2^ = 0.7633, *P* ≤ 0.0102, where *x* = years and *y* = eggs/gram of soil).

In field F, the number of eggs declined from 1.60 to 0.06 (*P* ≤ 0.05) while the number of cysts did not change significantly. The number of eggs per cyst declined from 6.1 to 0.4 (*P* ≤ 0.05) (Table 2). A regression line fitted through all sampling points indicated a yearly decline in eggs of 26.8% (*y* = 0.39149e − 0.46749*x*, *r*^2^ = 0.797384, *P* ≤ 0068, where *x* = years and *y* = eggs/gram of soil).

Populations in G were barely at the detection level when first sampled (Table 2). A decline in eggs and eggs per cyst was not detected during the course of the study. There was an increase in cysts at 5.1 years when the field was in tomatoes (*P* ≤ 0.05).

Field H had detectable levels of cysts but not eggs when first sampled shortly after sugarbeets were harvested (Table 2). Sampling shortly afterwards at 0.3 years produced 0.67 eggs and 0.006 cysts per gram of soil and 6.4 eggs per cyst. These numbers declined over the next six years to non-detectable levels of eggs and of eggs per cyst (*P* ≤ 0.05). A linear regression line fitted through all but the initial sampling point indicated a yearly decline in eggs of 18.9% per year (*y* = −0.0899*x* + 0.4753, *r*^2^ = 0.5869, *P* ≤ 0.0757, where *x* = years and *y* = eggs/gram of soil).

Sugarbeets had been recently harvested from Field I when it was first sampled. At that time, it had the highest number of eggs per gram and eggs per cyst of any of the fields sampled. Subsequently, it was fallow for three years of the study and in corn and sugarbeets for one year each (Table 1). There was an overall decline from 9.59 eggs and 0.204 cysts to 1.17 eggs and 0.151 cysts per gram of soil (*P* ≤ 0.05) (Table 2). The number of eggs per cyst declined from 25 to 4.7 during the same period (*P* ≤ 0.05). Populations did not increase during the time it was in sugarbeets (2.3 to 3.3 years). A regression line fitted through all sampling points indicated a yearly decline in eggs of 21.1% (*y* = −1.9965*x* + 9.4488, *r*^2^ = 0.9223, *P* ≤ 0.0094, where *x* = years and *y* = eggs/gram of soil).

Field J was planted to tomatoes at the time of first sampling. It had been in sugarbeets the previous year for the first time in 20 years, when the grower reported a serious problem with SBCN. Following tomatoes, corn was planted three years in a row, then sugarbeets and wheat (Table 1). During the course of the study, the number declined from 1.51 eggs and 0.188 cysts to 0.32 eggs and 0.074 cysts per gram of soil (Table 2). Eggs per cyst declined from 5.1 to 1.9. A regression line fitted through all sampling points indicated a yearly decline in eggs of 24.8% (*y* = 0.41454e − 0.43177*x*, *r*^2^ = 0.981131, *P* ≤ 0.0095, where *x* = years and *y* = eggs/gram of soil).

At the time of first sampling, no other plant parasitic nematodes were detected in six of the 10 fields. Three fields contained *Pratylenchus* sp., two each *Meloidogyne* sp. and *Xiphinema* sp., and one each *Tylenchorhynchus* sp. and *Helicotylenchus* sp (Table 1).

Fields in the Collegeville area (A to H) were either a silty clay or a silty clay loam, with levels of organic matter ranging from 1.4% to 2.4%. In the Eight Mile Road area, fields (I to J) were a loam with organic matter ranging from 3.9% to 6.2% (Table 3).

**Table 3:** Physical characteristics of soils in fields sampled in the study.

		**Percent**										
**Field location**	**Soil type**	**Sand**	**Silt**	**Clay**	**OM^a^**	**SP**	**pH**	**EC millimhos/cm**	**Ca milliequivalents/l**	**Mg milliequivalents/l**	**Na milliequivalents/l**	**SAR**
A	silty clay loam	15	48	37	2.0	46	7.0	0.81	3.1	2.9	2.1	1
B	silty clay	11	44	45	2.1	53	7.2	0.62	2.5	1.9	1.8	1
C	silty clay	12	47	41	2.4	55	7.3	0.61	2.4	2.1	1.6	1
D	silty clay	14	45	41	1.8	50	6.9	0.80	3.4	2.3	2.3	1
E	silty clay	12	44	44	1.8	48	7.0	0.67	3.0	1.8	1.9	1
F	silty clay	15	44	41	1.5	46	7.1	0.69	3.1	2.3	1.5	1
G	silty clay loam	14	47	39	1.5	45	6.2	0.59	2.0	1.8	2.1	2
H	silty clay	13	42	45	1.4	44	6.1	0.65	2.3	2.1	2.1	11
I	loam	35	44	21	6.2	47	7.4	0.73	3.7	2.3	1.3	1
J	loam	37	37	25	5.2	44	7.3	0.58	2.9	1.7	1.2	1

a OM = Stable Organic Matter, SP = Saturation Percentage, EC = Electrical Conductivity, SAR = Sodium Absorption Ratio

## Discussion

A significant finding of this study is that San Joaquin County’s rates of population decline are slower than they are in the Imperial and Ventura Counties. This validates grower experience that longer rotations between crops of sugarbeet are required. A slower decline rate necessitates a longer rotation between crops of sugarbeet impacting the frequency with which the crop can be grown. Additionally, we found that reproduction of SBCN on tomatoes, that had been previously shown in greenhouse trials, also occurs in grower fields.

The first recorded nematode pathogen of sugarbeets was SBCN, and it remains an important pathogen (Altman and Thomason, 1971; Cooke, 1993; Roberts and Thomason, 1981). SBCN is common and a significant problem in most areas of the world where sugarbeets are grown (Cooke, 1993). It is considered the third most important plant-parasitic nematode in the world (Bernard et al., 2017; Sasser and Freckman, 1987). The SBCN has hosts in a range of plant families; Steele (1965) investigated approximately 200 hosts in 98 genera from 23 out of 49 families. Of the agronomic crops that are known hosts, most occur within the Chenopodiaceae (sugarbeet, fodder beet, red beet, mangold, and spinach) and the Cruciferae (cabbage, kale, brussels sprout, broccoli, cauliflower, turnip, kohlrabi, mustard, and radish).

Over a period of several years, Roberts et al. (1981) sampled three fields infested with SBCN in the Imperial Valley and one on the Oxnard plain of California at 0 to 30- and 30 to 60-cm depths from two to five sites sampled in each field, with eight subsamples per sampling site. In their study, populations declined at the rate of 49% to 80%, leading to predictions of three- to four-year rotations for numbers to drop below the damage threshold of one to two eggs/gram of soil in these areas (Roberts and Thomason, 1981). In contrast, growers in the “spring harvest” area of the San Joaquin Valley reported needing eight to 10 years of rotation for SBCN to drop below damaging levels (F. Kegel, personal communication).

In England, Jones (1956) found the rate of decline of SBCN eggs to be 33% to 50% per year. In the Netherlands, Ouden (1956) obtained yearly egg decline rates for SBCN of 38% to 66%. In England, Moriarty (1963) reported SBCN egg decline rates ranging from 36% to 60% per year in one study and from 37% to 67% in another (Moriarty, 1961). In the current study, yearly rates of population decline could be measured in nine of the 10 fields examined and ranged from 12.2% to 34.6%. Rates of population decline were similar for “spring” and “fall harvest” areas.

Weed management during rotations could account at least partially for the apparent slow rate of decline of SBCN in this study. In the Imperial Valley and Oxnard Plain of California, it is common for two or even three rotation crops to be grown within a single year, with weed management being conducted for each crop. In the San Joaquin Valley, on the other hand, one crop per year during the spring and summer with a prolonged period of “fallow” during the fall and winter is common. During this “fallow” period, weed management is difficult because of the prolonged rainy season. Primary weeds in this area, which are reported hosts of SBCN, include yellow mustard (*Brassica* sp.), and shepherd’s purse (*Capsella burs-pastoris*). The population increase — occurring in field D during a year in which no crop was grown — could have occurred on weedy hosts. Longer rotations may also be required because overwintering of sugarbeets in “spring harvest” areas could lead to higher populations at harvest that would result in longer rotations being required.

Suppressive soils (loosely defined as fields that should have nematode problems but do not) have intrigued nematologists for many years (Westphal, 2005). Research has found that soils suppressive to SBCN frequently have one or more species of nematode parasitic fungi. Jaffee et al. (1991) found that *H. rhossiliensis*, which is parasitic on juveniles of SBCN, was present in California sugarbeet fields (including 80% of fields sampled in San Joaquin County), as was the ring trapping fungus *Arthrobotrys dactyloides*. Fungi parasitic on cysts and eggs of SBCN — including *Hyalorbilia oviparasitica*, *Dactylella oviparasitica*, and *Brachypyoris oviparasitica* — are also common in fields with a history of growing sugarbeets (Chen et al. 2021). These fungi could be a factor in the rates of decline seen in the fields studied. Tedford et al. (1993) suggest that the parasitic fungal populations are self-regulating in that they maintain a level below that which will eradicate the nematode population, thus ensuring that a continuous food source will be available.

Soil properties have been shown to affect nematode reproduction. For example, working with two soil types, Santo and Bolander (1979) demonstrated that *Meloidogyne hapla* reproduced best in a sandy loam soil while SBCN reproduced best in a silt loam. Cooke (1991) found that damage to sugarbeets was less in organic soils than in mineral soils. Fields in the present study were selected because growers had experienced significant damage to sugarbeets from SBCN. The fields in the Roberts et al. (1981) study on SBCN decline rates in southern California were both finer- and coarser-textured than in our study. Their fields had a clay content as high as 58.6% in one case and a sand content as high as 53.1% in another. The highest clay content in our fields was 45%, and the highest sand content was 37%. Although there are statistically significant differences in reproduction on various soil types, SBCN reproduces well enough in a wide range of soil types to cause significant economic damage to sugarbeet.

SBCN can produce more than 600 eggs/cyst under laboratory conditions (Raski, 1949), with several hundred per cyst not being unusual (Caswell and Thomason, 1991). Throughout the course of this study, on average, relatively few eggs were recovered per cyst. This is consistent with other samples processed from the San Joaquin Valley area over a period of years (B. Westerdahl, personal communication).

In fields that contained cabbage in rotation, the crop was typically planted in March and harvested in May or June, allowing minimal time for nematode reproduction to occur. Populations in Fields A and B declined in spite of cabbage in rotation.

Tomatoes are a host for SBCN in greenhouse trials (Lear and Miyagawa, 1972; Griffin and Waite, 1982). In this study, SBCN populations did not increase every time a field was planted to tomatoes, but increases were seen on three occasions, each time in a different field (A, B, and C).

The apparent population increase which occurred in field H several months following harvest of the sugarbeets could have been due to maturation of cysts on roots remaining in the soil following harvest, as has been demonstrated in microplot trials (Gardner and Caswell-Chen, 1997).

A shovel was utilized for sampling because soil types in this sugarbeet growing area typically contain substantial amounts of clay and silt (Table 3) and are difficult to sample with soil probes. Sampling was confined to the top 30 cm for two reasons: (i) because of soil texture, and (ii) because a previous study by Roberts et al. (1981) — in which samples from 0 to 30- and 30 to 60-cm depths were compared — indicated that highest numbers of cysts and eggs were found at the shallower depth.

This study demonstrates that the need for longer rotations in the San Joaquin Valley sugarbeet growing area is likely due to a combination of factors: (i) an apparent slower rate of population decline on rotation crops, (ii) weed hosts during rotations allowing populations to increase, (iii) population increases on tomatoes, and (iv) continued reproduction during winter months on overwintered sugarbeets. The possibility of a lower damage threshold in this area of California should be examined in future research.

A non-linear critical point model developed by Seinhorst (1965) has been used to relate yield of sugarbeets to initial populations of SBCN at the time of planting (Cooke and Thomason, 1979; Greco, Brandonisio and de Marinis, 1982; Cooke, 1984). Intraspecific competition among nematodes results in decreased damage per nematode as density increases. The model predicts that lower initial populations will result in higher populations at harvest than beginning with higher populations, leading to the subsequent need for increased lengths of rotation. Production losses owing to SBCN vary but can be as high as 60% of the crop (Ghaemi et al., 2020).

Based on crop rotation programs developed in England (Cooke, 1991) to reduce losses on sugarbeet due to SBCN, in California, processors, growers, County Agricultural Commissioners, and the University of California developed a program in which sugarbeets cannot be planted in non-infested fields more than two consecutive years and not more than four out of 10 years. In infested fields, sugarbeets can be grown only once every four years (Roberts and Thomason, 1981). Field infestation is monitored by an intensive sampling program conducted by processors and enforced through their contracts with growers. The success of this program is due to the natural decline of SBCN in the absence of host plants. Slower rates of decline in a region result in a longer length of rotation between sugarbeet crops, which could impact the economics of using sugarbeets for production of biofuel.

Interest in the use sugarbeets to produce bioethanol is increasing. The results of this study indicate that the potential impact of SBCN and other pests on the economics of bioethanol production should be assessed. Jiménez-Islas et al. (2021) conducted a bibliometric analysis of the Web of Science database to identify research related to sugarbeet as a biofuel. From 2003 to 2019, an exponential growth of publications was found, with Germany and the United States being the countries with the highest rates of increase. Growth can be attributed to the development of renewable energy and the relevance of global warming, energy security, and laws that promote clean energy.

Several studies have provided estimates of the amount of bioethanol that can be produced from sugarbeets. A 2006 USDA study calculated the yield of ethanol from the sucrose in a sugarbeet was 103.5 liters per 907 kg of root (wet weight) (Panella and Kaffka, 2010). In North Dakota, research suggests that 100.3 liters of ethanol can be produced from each 907 kg of sugarbeets (Farm Progress, 2010). A University of California, Davis study estimated 126.8 liters of ethanol could be produced from 907 kg of sugarbeets. (Zhang et al., 2011). A study by Iowa State University estimates 99.9 liters of ethanol could be produced from 907 kg of sugarbeets. Average sugarbeet yields in the United States are 49,307 kg/ha, with approximately 9,525 kg of sugar being produced per ha (Spreckels Sugar, 2012). Using an average estimate of 107.6 liters of ethanol per 907 kg of root and 49,307 kg of root/ha, this would yield an estimated 5,844 liters/ha of ethanol from a ha of sugarbeets.
